# Screening for Mild Cognitive Impairment in Parkinson's Disease: Comparison of the Italian Versions of Three Neuropsychological Tests

**DOI:** 10.1155/2015/681976

**Published:** 2015-11-08

**Authors:** Angela Federico, Alice Maier, Greta Vianello, Daniela Mapelli, Michela Trentin, Giampietro Zanette, Alessandro Picelli, Marialuisa Gandolfi, Stefano Tamburin

**Affiliations:** ^1^Department of Neurological and Movement Sciences, University of Verona, Piazzale Scuro 10, 37134 Verona, Italy; ^2^Department of General Psychology, University of Padova, Via Venezia 8, 35100 Padua, Italy; ^3^Human Inspired Technologies Research Center, University of Padova, Via Venezia 8, 35100 Padua, Italy; ^4^Neurology Unit Pederzoli Hospital, Via Monte Baldo 24, 37019 Peschiera del Garda, Italy; ^5^Neuromotor and Cognitive Rehabilitation Research Centre, University of Verona, Piazzale Scuro 10, 37134 Verona, Italy

## Abstract

Mild cognitive impairment (MCI) is frequent in Parkinson's disease (PD). Recently proposed criteria for MCI in PD (PD-MCI) indicate level I diagnosis based on abbreviated assessment and level II based on comprehensive neuropsychological evaluation. The study explored the sensitivity and specificity of the Italian versions of three neuropsychological tests for level I diagnosis of PD-MCI. We recruited 100 consecutive PD patients. After screening for inclusion criteria, 43 patients were included. The sensitivity and specificity of the Mini Mental State Examination (MMSE), the Montreal Cognitive Assessment (MoCA), and the Addenbrooke's Cognitive Examination Revised (ACE-R) in comparison to level II diagnosis of PD-MCI were examined. PD-MCI was diagnosed (level II) in 51% of patients. Disease duration was significantly longer and PD motor scales were more severely impaired in MCI group. The receiver-operator characteristics curve documented nonsignificant difference in the performance of the three tests, with slight advantage of MMSE (corrected data). The time of administration favored MMSE. In Italian-speaking PD patients, MMSE might represent a good screening tool for PD-MCI, because of the shorter time of administration and the performance comparable to those of MoCA and ACE-R. Further studies are needed to validate the new PD-MCI criteria across different languages and cultures.

## 1. Introduction

Cognitive impairment is frequent in Parkinson's disease (PD) [1], and the spectrum of cognitive dysfunction ranges from mild cognitive impairment (MCI) to PD dementia (PD-D) [2, 3]. The diagnosis of PD-D may to some extent be straightforward [4], but recognizing MCI in PD (PD-MCI) is more difficult. Cognitive deficits may occur early in PD course, and they can be documented in up to a quarter of newly diagnosed PD patients [5]. The biological validity of PD-MCI as a clinical entity is supported by converging morphological, functional neuroimaging, neurophysiological, genetic, and cerebrospinal fluid and histological data showing an association between a number of neuropathophysiological variables and cognitive impairment or cognitive decline in nondemented PD patients [2].

Identifying PD-MCI is clinically important, as these patients appear to be at increased risk for developing PD-D [6], and they often present functional impairment and have worse quality of life [2]. In the rehabilitation setting, recognizing PD-MCI is very important, in that it may negatively influence the outcome in patients undergoing motor rehabilitation. Moreover, PD-MCI may itself represent a target for cognitive training [7, 8], pharmacological treatment [9], or their combination.

A task force of the Movement Disorder Society (MDS) has recently delineated diagnostic criteria for PD-MCI [10]. These criteria indicate a two-step process with level I (possible PD-MCI) based on abbreviated assessment and level II diagnosis based on comprehensive neuropsychological evaluation permitting MCI subtyping [10], but they need to be validated, as well as the proposed neuropsychological scales and tests. A very recent study explored these criteria in a group of PD patients and the accuracy of three neuropsychological screening tests and found that none of them provided good combined sensitivity and specificity for PD-MCI [11]. For most of the neuropsychological tests, translation and validation across different languages and cultures are lacking, and this may represent a problem when assessing PD-MCI with level I criteria and a possible source of error when transferring data from a given population/language to other ones.

The present study was aimed to explore the sensitivity and specificity of the Italian versions of three neuropsychological tests for level I diagnosis of PD-MCI, namely, the Mini Mental State Examination (MMSE) [12], the Montreal Cognitive Assessment (MoCA) [13], and the Addenbrooke's Cognitive Examination Revised (ACE-R) [14], for all of which an Italian translation and validation exist [15–18]. Data from the three screening neuropsychological tests were compared to those from full neuropsychological testing (level II) [10], which represent the* gold standard* for MCI diagnosis.

## 2. Materials and Methods

### 2.1. Subjects

Our population sample was a group of 100 consecutive Italian PD patients. The study was carried out in accordance with the principles of the Declaration of Helsinki as revised in 2001 and approved by local ethics committee. All patients gave signed informed consent prior to inclusion in the study. Inclusion criteria were (1) diagnosis of PD based on the UK PD Brain Bank Criteria [19]; (2) absence of PD-D [4]; (3) no other possible causes for cognitive impairment (e.g., delirium, stroke or cerebrovascular disease, head trauma, metabolic abnormalities, and adverse effects of medication); (4) no other PD-associated comorbid conditions (e.g., marked motor impairment, severe or unpredictable motor fluctuations and/or dyskinesia, severe anxiety, excessive daytime sleepiness, or psychosis) that may have significantly influenced cognitive testing [10].

Depression was assessed with the Beck Depression Inventory II (BDI-II) [20] with a cutoff of 14 for the presence of mild depression and a cutoff of 28 for severe depression [21]. Depression was not considered an exclusion criterion, except if severe (i.e., patients with a BDI-II score >28 were excluded), because it may be found in around 35% of PD patients [22] and including PD patients with mild to moderate depression would have resulted in a more real-life scenario. The severity of PD motor symptoms and related impairment and disability was measured with the Modified Hoehn and Yahr Staging Scale [23] and the Unified Parkinson's disease rating scale [24]. Total daily levodopa equivalent dose was calculated for each patient [25].

**Figure 1:**
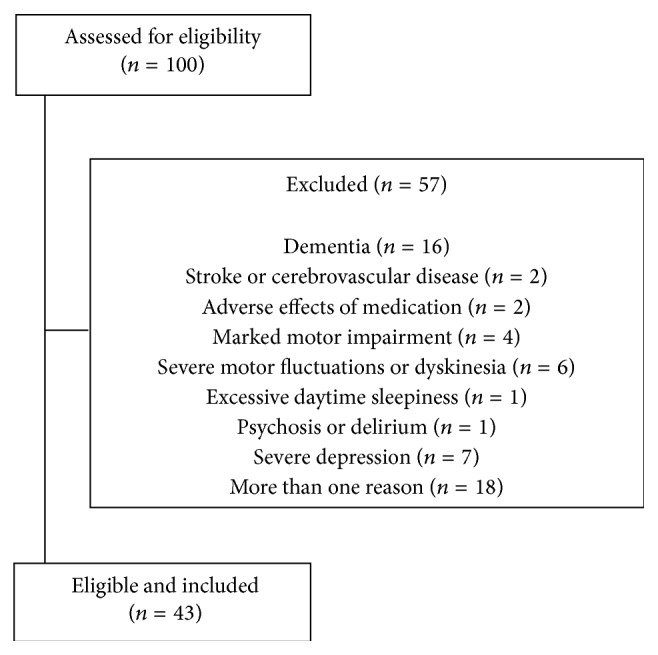
Flow diagram of the study and reasons for patients' exclusion.

After screening for inclusion criteria (Figure 1), 43 patients (27 males, 16 females, mean age 68.2 ± 9.2, range 44–88; mean education 8.5 ± 2.9 years, range 4–13) were included in the study. Demographic and clinical characteristics of patients are reported in Table 1.

### 2.2. Neuropsychological Assessment

All patients underwent the Italian versions of MMSE, MoCA, and ACE-R and a full neuropsychological testing, which were performed by different expert neuropsychologists, who were blinded to each other's results, on separate days at a similar time of the day, and with the patient in the ON state. Given overlapping items, the order of administration of the three screening tests was pseudorandom to avoid bias in performance related to fatigue, learning, or other effects secondary to order [11]. Since the ACE-R contains all the items of the MMSE, the common items were not administered twice. The time taken for administering each screening test and full neuropsychological testing was measured in each patient.

Full neuropsychological testing included at least two types of neuropsychological testing for each of the five following cognitive domains [10].* Attention and working memory* were examined with four tests, namely, digit span, a subtest of the Wechsler memory scale [26], interference memory task (10 sec and 30 sec) based on the Brown-Peterson paradigm [27, 28], and trail making test (TMT) part A [29].* Executive function* was explored with four tests, namely, TMT part B [29], frontal assessment battery [30], phonemic fluency test, and clock drawing test, the latter two being subtests of the short neuropsychological examination version 2 (ENB-2) [31].* Language* was examined with four tests, namely, the short form of the Boston naming test [32] and three specific subtests of the neuropsychological examination of aphasia [33].* Memory* was explored with four tests, namely, Rey's auditory verbal learning test (immediate recall, delayed recall) [34], and two prose recall subtest (immediate recall, delayed recall) derived from ENB-2 [31].* Visuospatial function* was examined with two tests, namely, Benton's judgment of line orientation [35] and the geometrical figures copying test, a subtest of the mental deterioration battery [36].

The impairment on basic activities of everyday life (BADL) and instrumental activities of everyday life (IADL) was explored with specific questionnaires [37, 38].

### 2.3. PD-MCI Diagnosis

The diagnosis of PD-MCI was made according to the MDS Task Force level II criteria [10]. They included (1) gradual decline, in the context of established PD, in cognitive ability reported by either the patient or informant or observed by the clinician, consisting of at least 1 item of the IADL scale; (2) cognitive deficits that are not sufficient to interfere significantly with functional independence, although subtle difficulties on complex functional tasks may be present, as documented by normal BADL scale; (3) impairment in at least two neuropsychological tests, represented by either two impaired tests in one cognitive domain (single-domain PD-MCI) or one impaired test in two different cognitive domains (multiple-domain PD-MCI). Impaired performance on a neuropsychological test was defined as a score that was at least 1.5 standard deviations (SDs) below the age-adjusted mean from normative data [11]. According to the MDS Task Force criteria, significant decline on serial cognitive testing or from estimated premorbid level may be used instead of normative data [10], but we did not use these alternative criteria, because the former would have required repeated full neuropsychological testing with the risk of learning bias and because of the difficulties found in applying the latter (see Section 4) [11].

### 2.4. Statistical Analysis

All tests were carried out with the IBM SPSS version 20.0 and the Stata 11.0 statistical packages. The normality of variable distribution was analyzed with the Skewness-Kurtosis test. Continuous variables were explored with ANOVA and post hoc *t*-test with Bonferroni's correction. Homogeneity of variance was analyzed with Levene's test. The data were transformed (logarithmic transformation) before submitting them to ANOVA in case of an inequality in the variances. The nonparametrical Mann-Whitney *U* test was applied in case the distribution was not normal. Pearson's *χ* ^2^ test with Yates' correction for continuity was applied to dichotomous variables. Sensitivity and specificity of the MMSE (raw score and score corrected for age, sex, and education), MoCA (raw and corrected score), and ACE-R were calculated across all possible cutoff scores below which an individual would be classified as having PD-MCI. The area under the receiver-operator characteristics (ROC) curve (AUC) was calculated and compared across the three tests and the AUC 95% confidence intervals (CIs) were generated. *p* < 0.05 (two-tailed) was taken as the significance threshold for all the tests.

## 3. Results

According to the MDS Task Force level II criteria [10], PD-MCI was diagnosed in 22 patients (51%). Eight out of the 22 (36%) PD-MCI patients were classified as single-domain MCI, with five of them showing impairment in executive function and three with impaired memory. The other 14 patients (64%) were classified as multiple-domain MCI. Among multiple-domain MCI cases, attention and working memory was impaired in 9 patients, executive function in 14, memory in 8, language in 2, and visuospatial function in 1. Demographic and clinical variables according to the presence or absence of MCI and the MCI subtype (i.e., single-domain versus multiple-domain) are reported in Table 2. Disease duration was significantly longer in patients with MCI (12.8 ± 8.1 years) than in those without MCI (7.8 ± 5.3 years, *p* = 0.03; Table 2). PD motor and impairment scales were more severely impaired in MCI group (H-Y: 2.5 ± 0.6; UPDRS-III: 30.2 ± 8.4) than in patients without MCI (H-Y: 1.9 ± 0.7, *p* = 0.014; UPDRS-III: 23.3 ± 8.9, *p* = 0.02; Table 2). The other variables did not differ between the two groups. None of the demographic and clinical variables significantly differed according to the MCI subtype (Table 2).

### 3.1. Comparison between the Screening Tests

**Figure 2:**
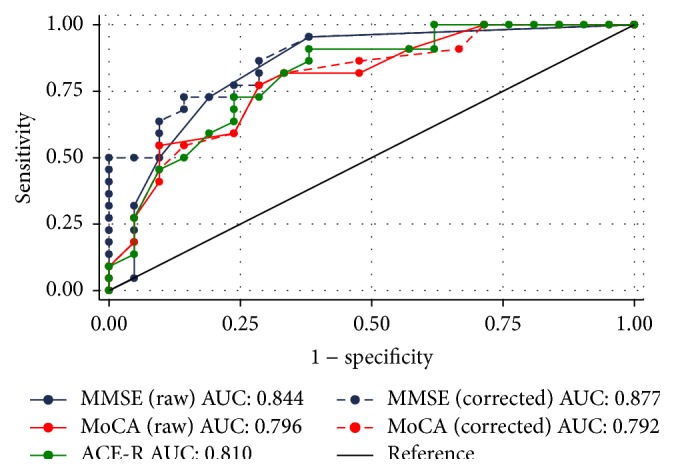
Receiver-operator characteristics (ROC) curves for the three screening tests (raw and corrected data).

The ROC curves for the three screening tests (raw and corrected data) are illustrated in Figure 2. The AUC was 0.84 (95% CI: 0.72–0.97) for the MMSE (raw data), 0.88 (95% CI: 0.78–0.98) for the MMSE (corrected data), 0.80 (95% CI: 0.66–0.93) for the MoCA (raw data), 0.79 (95% CI: 0.66–0.93) for the MoCA (corrected data), and 0.81 (95% CI: 0.68–0.94) for the ACE-R. None of the pair-wise comparisons between AUC estimates were statistically significant.

The sensitivity and specificity of the three tests for detecting PD-MCI across different cutoff scores are reported in Tables 3 and 4.

### 3.2. Screening Cutoff Values

For raw MMSE data, the lowest cutoff value with sensitivity >0.80 was 29.5 (sensitivity = 0.96, specificity = 0.62). When using corrected MMSE data, the lowest cutoff value with sensitivity >0.80 was 28.6 (sensitivity = 0.86, specificity = 0.71). For raw MoCA data, the lowest cutoff value with sensitivity >0.80 was 24.5 (sensitivity = 0.82, specificity = 0.67). When analyzing corrected MoCA data, the lowest cutoff value with sensitivity >0.80 was 25.5 (sensitivity = 0.82, specificity = 0.67). For ACE-R, the lowest cutoff value with sensitivity >0.80 was 86.0 (sensitivity = 0.82, specificity = 0.67).

### 3.3. Diagnostic Cutoff Values

For raw MMSE data, the highest cutoff value with specificity >0.80 was 28.5 (sensitivity = 0.73, specificity = 0.81). When examining corrected MMSE data, the highest cutoff value with specificity >0.80 was 28.0 (sensitivity = 0.73, specificity = 0.81). For raw MoCA data, the highest cutoff value with specificity >0.80 was 21.5 (sensitivity = 0.55, specificity = 0.90). When using corrected MoCA data, the highest cutoff value with specificity >0.80 was 22.5 (sensitivity = 0.55, specificity = 0.86). When analyzing ACE-R findings, the highest cutoff value with specificity >0.80 was 77.5 (sensitivity = 0.59, specificity = 0.81).

### 3.4. Timing for Administering Screening Tests and Full Neuropsychological Testing

The average time for the administration of the screening tests was 7.8 ± 1.4 min for MMSE, 12.3 ± 3.2 min for MoCA, and 18.4 ± 2.9 min for ACE-R. Full neuropsychological testing required 52.3 ± 7.1 min.

## 4. Discussion

We have explored the sensitivity and specificity of the Italian versions of three screening tests for recognizing PD-MCI in comparison to full neuropsychological testing. Our data documented that the performances of the three tests were similar and that they could achieve a limited trade-off between sensitivity and specificity, with a slight advantage of MMSE and the use of corrected data.

The screening tests we examined were chosen because, to the best of our knowledge, they were the only ones with the availability of a validated Italian version at the time when the study was designed. None of them could reach combined sensitivity and specificity >0.80 at any cutoff value. The analysis of ROC curves for the screening scales showed a larger AUC and the best sensitivity-specificity profile for the corrected MMSE score. In particular, a cutoff of 28.6 resulted in sensitivity = 0.86 and specificity = 0.71, while a cutoff of 28.0 was associated in sensitivity = 0.73, and specificity = 0.81. The other scales performed slightly worse, but the difference between the ROC curves was not significant.

A number of previous studies compared different screening tests for assessing cognitive functions and/or early cognitive deficit in PD patients [5, 39], with conflicting results in terms of the best profile of sensitivity and specificity between them. The use of MMSE as a screening instrument in PD has been challenged because it does not specifically test subcortical executive function, which is impaired early in PD patients [40]. Some studies documented that MMSE has low sensitivity in detecting MCI and cognitive impairment in PD [41, 42], in particular when compared to MoCA [39, 43–45]. At variance, other authors reported that MMSE might be useful in detecting cognitive deterioration in early PD [46]. Data on the use of ACE-R as a screening tool for PD-MCI are controversial [47], but a previous version was found to be a good test for evaluating MCI [48] and dementia [49, 50] in PD patients. A reason for these discrepancies might be that ACE-R includes an assessment by domains and its abilities may not be completely comparable to that of MMSE and MoCA, which represent true screening scales. Moreover, MMSE and ACE-R share some common items, and the total points of ACE-R (100 points) differ from that of MMSE and MoCA (30 points). However, the comparison of AUCs instead of cutoffs should have avoided the difference in total points among screening tests to represent a bias.

Comparison between the present results and those from most of previous studies is however difficult, because only a few of them used a comprehensive neuropsychological evaluation and standard criteria to detect and diagnose MCI. A couple of recent studies applied the new MDS criteria for MCI and yielded contrasting results, in that one documented limited sensitivity-specificity profile for both MMSE and MoCA [11], while the other reported high sensitivity of MoCA for predicting PD-MCI [51].

Other neuropsychological scales, such as the Mattis dementia rating scale [48, 52, 53], the Cambridge cognitive assessment [54], the cognitive linguistic quick test [55], the PD cognitive rating scale [56], and the SCOPA cognition [57], have been demonstrated to be helpful in exploring early cognitive decline in PD [10], but the absence of an Italian version impeded their exploration as a screening tool for PD-MCI in our PD patients sample. What is more, the long administration time of these scales (i.e., up to 25–45′) is not suitable for a screening procedure in the clinical setting.

Our data favor the correction for age, sex, and education when scoring MMSE, in that corrected MMSE data yielded a larger AUC and slightly better sensitivity-specificity profile than raw ones. At variance, correcting MoCA did not change the performance of the test. However, pair-wise statistical comparisons between ROC curves did not show any significant difference between them. In the clinical setting, MMSE correction is reasonable especially for older and less educated patients.

We recorded the time taken for administering the three screening scale, and this variable favored the MMSE (7.8 ± 1.4 min) compared to the MoCA (12.3 ± 3.2 min) and the ACE-R (18.4 ± 2.9 min). According to these combined figures (i.e., similar sensitivity-specificity profile, shorter time of administration), it is reasonable to prefer the use of MMSE in the setting of a busy clinic.

A number of factors may contribute to cognitive dysfunction in PD patients and lead to a false positive diagnosis of PD-MCI. All the possible contributing factors were considered and our strict inclusion criteria, which resulted in the exclusion of approximately half of the patients, should have reduced this bias. Drugs with possible effect on cognition represented an exclusion criterion, and the total LED was similar between patients with and without MCI. As a consequence, pharmacological effects should not have influenced our findings. Depression has been documented to be more frequent in PD-MCI patients in comparison to those without MCI [58], but this was not the case in our sample. We excluded only patients with severe depression according to the BDI-II, because mild to moderate depression is a common feature of PD and exclusion of all depressed patients might have resulted in a non-real-life scenario. We may argue that depression should not have been a bias factor in the present study.

PD patients with MCI had significantly longer disease duration and more severe motor impairment and disability, according to the H-Y and UPDRS-III scales. This finding is in accordance with some previous reports [58] but in contrast with other ones [11]. Differences in the sampling of PD patients across different studies, depending on different settings (e.g., primary care versus referral centre) or different populations, are the most likely reasons for this discrepancy.

The analysis of MCI subtypes indicated a prevalence of multidomain PD-MCI in comparison to single-domain. This finding is in accordance with previous reports using new MDS criteria [11, 59]. We could not document any difference in clinical variables between single- and multidomain PD-MCI patients, but the small samples might have impeded the recognition of small differences between the two groups. In accordance with previous studies [5, 7], we documented a high prevalence of executive alterations in our PD sample. This finding may stem from the use of four tests for this cognitive domain, which may have resulted in a higher likelihood of falling in two of them [60]. However, this potential bias effect seems not to be major, because the upper limit (maximum probability) for detecting impairment on a test was found to stabilize at two tests in the executive functions domain and did not increase with three or four tests [60].

When applying the MDS level II diagnostic criteria for PD-MCI [10], impairment on a neuropsychological test was defined as a score that was at least 1.5 SD below the age-adjusted normative data [11]. We avoided the use of the alternative criterion of a significant decline on serial cognitive testing [10], because of the lack of previous neuropsychological testing in the majority of our patients. For what concerns the other alternative criteria of a decline from estimated premorbid level [10], this was also not used for a number of reasons. They include the lack of any indication on how to use tests of premorbid intellectual functioning [10], the absence of a validated Italian version of the Wechsler test of adult reading [10], and the previous evidence of the ineffectiveness of the Italian version of the alternative national adult reading test for the estimation of premorbid reading ability [61]. In a previous study, the number of patients diagnosed as PD-MCI with level II criteria varied consistently (i.e., from 33% to 79%) by applying different criteria for impairment on a neuropsychological test [11], and this might represent an important source of uncertainty when applying level II criteria. Similarly, varying cutoff values for single tests had a large influence on the percentage of PD-MCI patients in the same population [62].

Limitations of the present study include the small sample and the high prevalence of PD-MCI. MCI was found in 51% patients in our PD sample, while cross-sectional studies documented that the prevalence of MCI ranges from 20 to 30% in PD [42, 58]. However, our sample is too small to provide a good approximation of the prevalence of the condition in the general population, and there may have been some bias due to the strict selection criteria. The present data should be confirmed in a larger PD patients group before generalizing our conclusions.

Another limitation is the absence of follow-up data. Serial testing of PD-MCI patients documented that a similar proportion of them might either progress to PD-D or revert to normal cognition (i.e., approximately 20%) after one year [63]. Reasons for this apparently paradoxical finding might include comorbidities, measurement errors, learning effects due to repeated neuropsychological testing, and improved cognition after initiation of symptomatic treatment [63], in addition to suboptimal treatment of motor symptoms at the time of first testing, motor fluctuations, or drug side effects.

BADL and IADL were evaluated with questionnaires [37, 38] that are not PD-specific, because, to the best of our knowledge, there is no Italian version of any disease-specific scale, such as the Parkinson's disease cognitive functional rating scale [64]. We think that this point does not represent a major bias, because the questionnaires were used to group patients as having PD-MCI or not and not to quantitatively measure impairment on BADL and IADL.

## 5. Conclusions

Our data might be helpful in the clinical and the neurorehabilitation setting, because cognitive impairment is common in PD, PD-MCI may progress to PD-D, and both these conditions may have a negative impact on function, quality of life, and caregiver burden [43]. Identification and intervention at the earliest stage of PD-MCI is a crucial unmet need for the overall care of PD patients [10]. MMSE might represent a good tool for screening cognition throughout all stages of PD, because of the short time of administration and the sensitivity-specificity profile comparable to those of MoCA and ACE-R. Follow-up serial testing might be necessary in case of confounding factors. Complete neuropsychological testing, however, still represents the* gold standard* for a diagnosis of PD-MCI.

Future studies should better explore the reliability of level I and level II MDS criteria for MCI and incorporate biomarkers of cognitive dysfunction [2, 10].

## Figures and Tables

**Table 1:** Demographic and clinical characteristics of the patients.

Pt	Sex	Age (y)	School (y)	Duration (y)	H-Y (1–5)	Treatment and daily dosage	Depression (yes/no)
1	F	44	12	5	1	PRX 4.5 mg, RAS 1 mg	No
2	M	46	8	15	3	LD 600 mg, APO 36 mg, PRX 3 mg	Yes
3	M	51	13	10	2.5	LD 1150 mg	No
4	F	52	11	14	2	APO 84 mg, CAB 9 mg	No
5	F	56	13	11	2.5	LD 500 mg, APO 48 mg, PRX 4.5 mg	Yes
6	F	57	13	10	2	LD 600 mg, APO 52 mg	No
7	M	60	12	8	3	LD 500 mg	Yes
8	M	60	8	20	3	LD 750 mg	Yes
9	M	61	8	5	1	RAS 1 mg	Yes
10	M	61	13	21	2.5	LD 950 mg	No
11	F	63	10	4	1.5	PRX 3 mg	Yes
12	F	64	5	2	1.5	LD 400 mg	Yes
13	F	65	5	10	2.5	LD 700 mg, PRX 1.5 mg, RAS 1 mg	No
14	F	67	13	10	2	LD 1000 mg, PRX 4.5 mg	No
15	M	67	10	5	1	LD 400 mg	No
16	M	67	8	1	1	None	Yes
17	M	68	8	3	1.5	LD 400 mg	Yes
18	M	68	8	22	2.5	LD 975 mg	Yes
19	M	68	10	21	3	LD 1250 mg	Yes
20	M	68	8	23	2.5	LD 1150 mg	No
21	M	69	8	24	3	LD 950 mg	Yes
22	M	69	5	4	3	LD 850 mg	No
23	M	70	10	24	4	LD 1200 mg	Yes
24	F	70	3	10	1.5	LD 800 mg	No
25	M	72	8	4	1	LD 350 mg, ROP 8 mg	No
26	F	72	12	12	2.5	LD 550 mg, APO 42 mg	No
27	M	72	8	12	2.5	LD 1250 mg	No
28	F	73	5	5	1	LD 250 mg, SEL 10 mg	No
29	M	73	13	7	3	LD 1250 mg, ROT 10 mg	No
30	M	74	8	4	3	LD 700 mg, PRX 3 mg	No
31	M	75	8	6	3	LD 600 mg, ROP 16 mg	No
32	M	75	5	3	1	ROP 16 mg, SEL 10 mg	No
33	M	75	5	20	3	LD 1100 mg, ROP 12 mg	No
34	M	75	5	10	2.5	LD 800 mg, PRX 4.5 mg	Yes
35	F	75	5	5	2	LD 300 mg, ROP 16 mg	No
36	M	76	8	20	2.5	LD 1150 mg, PRX 3 mg	No
37	M	76	6	10	2	LD 900 mg, PRX 3 mg	No
38	M	76	5	25	3	LD 1250 mg	No
39	F	76	8	8	2	LD 1000 mg	Yes
40	M	76	13	11	2	LD 750 mg	Yes
41	F	79	5	4	2	LD 300 mg	No
42	F	83	5	4	2	LD 500 mg	Yes
43	F	88	4	1	1.5	LD 400 mg	No
Average		**68.2**	**8.3**	**10.5**	**2.2**		
SD		**9.3**	**3.0**	**7.3**	**0.8**		

Pt: patient; school: education (years); duration: disease duration (years); H-Y: Modified Hoehn and Yahr Staging Scale (range 1–5); SD: standard deviation; APO: apomorphine; CAB: cabergoline; LD: levodopa (dosage corrected according to the eventual use of COMT-inhibitors); PRX: pramipexole; RAS: rasagiline; ROP: ropinirole; ROT: rotigotine; SEL: selegiline.

**Table 2:** Characteristics of patients, according to the diagnosis and subtype of PD-MCI (MDS Task Force level II criteria).

	No PD-MCI (*n* = 21)	PD-MCI (*n* = 22)	*p*	Single-domain PD-MCI (*n* = 8)	Multiple-domain PD-MCI (*n* = 14)	*p*
Age	67.5 ± 11.2	68.9 ± 7.2	n.s.	65.5 ± 7.7	70.8 ± 6.2	n.s.
Sex (M/F)	12/9	15/7	n.s.	7/1	8/6	n.s.
School (y)	8.7 ± 3.1	8.3 ± 2.8	n.s.	9.3 ± 2.6	7.6 ± 2.8	n.s.
Duration (y)	7.8 ± 5.3	12.8 ± 8.1	0.03	13.1 ± 8.6	11.4 ± 8.1	n.s.
H-Y (1–5)	1.9 ± 0.7	2.5 ± 0.6	0.014	2.3 ± 0.8	2.6 ± 0.5	n.s.
UPDRS-III (0–108)	23.3 ± 8.9	30.2 ± 8.4	0.02	27.5 ± 10.2	31.5 ± 9.0	n.s.
Treatment						
LD (yes/no)	17/4	20/2	n.s.	7/1	13/1	n.s.
DA (yes/no)	12/9	7/15	n.s.	2/6	5/9	n.s.
MAO-I (yes/no)	4/17	1/21	n.s.	0/8	1/13	n.s.
Total LED (mg)	821 ± 413	889 ± 394	n.s.	893 ± 439	888 ± 384	n.s.
Depression (yes/no)	8/13	9/13	n.s.	3/5	6/8	n.s.

School: education (years); duration: disease duration (years); H-Y: Modified Hoehn and Yahr Staging Scale (range 1–5); UPDRS-III: Unified Parkinson's Disease Rating Scale part III (range 0–108); LD: levodopa; DA: dopamine agonist; MAO-I: monoamine oxidase inhibitors; LED: levodopa equivalent dose (daily).

**Table 3:** Sensitivity and specificity of MMSE and MoCA for detecting PD-MCI at different cutoff values.

Cutoff	Sensitivity	Specificity
MMSE		
Raw data		
<24.5	0.05	0.95
<25.5	0.23	0.95
<26.5	0.32	0.95
<27.5	0.50	0.90
<28.5	0.73	0.81
<29.5	0.96	0.62
Corrected data		
<22.5	0.05	1.00
<23.2	0.09	1.00
<23.7	0.14	1.00
<24.3	0.18	1.00
<25.0	0.27	1.00
<25.2	0.32	1.00
<25.4	0.36	1.00
<25.7	0.41	1.00
<26.0	0.46	1.00
<26.2	0.50	1.00
<26.3	0.50	0.95
<26.4	0.50	0.91
<26.6	0.55	0.91
<26.8	0.59	0.91
<27.0	0.64	0.91
<27.2	0.68	0.86
<27.6	0.73	0.86
<28.0	0.73	0.81
<28.4	0.77	0.76
<28.6	0.86	0.71
<29.4	0.95	0.62

MoCA		
Raw data		
<15.0	0.05	1.00
<16.5	0.09	1.00
<17.5	0.18	0.95
<19.0	0.27	0.95
<20.5	0.41	0.90
<21.5	0.55	0.90
<22.5	0.59	0.76
<23.5	0.77	0.71
<24.5	0.82	0.67
<25.5	0.82	0.52
<26.5	0.91	0.33
<27.5	1.00	0.29
<28.5	1.00	0.14
<30.0	1.00	0.00
Corrected data		
<16.0	0.05	1.00
<17.5	0.09	1.00
<18.5	0.18	0.95
<20.0	0.27	0.95
<21.5	0.46	0.90
<22.5	0.55	0.86
<23.5	0.59	0.76
<24.5	0.77	0.71
<25.5	0.82	0.67
<26.5	0.86	0.52
<27.5	0.91	0.33
<28.5	1.00	0.29
<29.5	1.00	0.05

MMSE: Mini Mental State Examination; MoCA: Montreal Cognitive Assessment; PD-MCI: mild cognitive impairment in Parkinson's disease.

**Table 4:** Sensitivity and specificity of ACE-R for detecting PD-MCI at different cutoff values.

Cutoff	Sensitivity	Specificity
<62.5	0.05	1.00
<66.5	0.09	1.00
<69.5	0.14	0.95
<73.5	0.27	0.95
<75.5	0.46	0.90
<76.5	0.50	0.86
<77.5	0.59	0.81
<78.5	0.64	0.76
<80.0	0.68	0.76
<82.5	0.73	0.76
<84.5	0.73	0.71
<86.0	0.82	0.67
<87.5	0.86	0.62
<89.0	0.91	0.62
<90.5	0.91	0.43
<91.5	0.91	0.38
<92.5	1.00	0.38
<93.5	1.00	0.29
<94.5	1.00	0.24
<95.5	1.00	0.19
<96.5	1.00	0.14
<97.5	1.00	0.09
<98.5	1.00	0.05
<100.0	1.00	0.00

ACE-R: Addenbrooke's Cognitive Examination Revised; PD-MCI: mild cognitive impairment in Parkinson's disease.
